# Delivery of streptomycin to the rat colon by use of electrospun nanofibers

**DOI:** 10.1038/s41598-022-25769-z

**Published:** 2022-12-13

**Authors:** Priscila R. Guerra, Fatemeh Ajalloueian, Shaodong Wei, Katja Ann Kristensen, Martin Iain Bahl, Anja Boisen, Tine Rask Licht

**Affiliations:** 1 National Food Institute, Technical University of Denmark, 2800 Kgs. Lyngby, Denmark; 2 Department of Health Technology, Technical University of Denmark, 2800 Kgs. Lyngby, Denmark

**Keywords:** Biological techniques, Microbiology, Molecular biology, Systems biology, Nanoscience and technology

## Abstract

Drug-loaded electrospun nanofibers are potential drug carrier systems that may optimize disease treatment while reducing the impact on commensal microbes. The feasibility of streptomycin-loaded pullulan nanofibers fabricated from a green electrospinning procedure using water as the solvent was assessed. We conducted a rat study including a group treated with streptomycin-loaded nanofibers (STR-F, n = 5), a group treated with similar concentrations of streptomycin in the drinking water (STR-W, n = 5), and a non-treated control group (CTR, n = 5). Streptomycin was successfully loaded into nanofibers and delivered by this vehicle, which minimized the quantity of the drug released in the ileal compartment of the gut. Ingested streptomycin-resistant *E. coli* colonized of up to 10^6^ CFU/g feces, revealing a selective effect of streptomycin even when given in the low amounts allowed by the nanofiber-based delivery. 16S amplicon sequencing of the indigenous microbiota revealed differential effects in the three groups. An increase of *Peptostreptococcaceae* in the cecum of STR-F animals may indicate that the fermentation of nanofibers directly or indirectly promoted growth of bacteria within this family. Our results elucidate relevant properties of electrospun nanofibers as a novel vehicle for delivery of antimicrobials to the large intestine.

## Introduction

Antibiotics are crucial to control and prevent infections, but may also disturb non-target bacteria in the gut lumen^1–3^. Negative effects of antimicrobial therapies include disruption of the commensal intestinal microbiota as well as development of drug-resistant bacteria^3,4^.

Developments within biomaterials technologies have provided advanced drug carriers, such as nanofibers, which might help overcome challenges with therapeutic drug efficacy, reduce the risk of developing antimicrobial resistance^5,6^, and potentially minimize the impact on the gut microbiota.

Interestingly, drug delivery by nanostructured carriers has demonstrated relevant features such as efficient inhibition of bacterial growth and sustained release^7^. Moreover, drug-delivery systems based on polysaccharides, particularly pullulan, are attracting more attention since they are non-toxic, stable, biocompatible, and biodegradable^8–11^. Electrospinning, which is a simple and fast nanofiber-forming system, is moving forward in creating new kinds of nanostructures including multilayer sheets^12,13^, nano-hybrids^14,15^, and core–shell nanofibers^14,16^. Different potential electrospinning strategies for manipulating drug release behaviors for an improved therapeutic effect have recently been reviewed^17,18^. Against the broad use of electrospun nanofibers for drug delivery applications, several introduced electrospun nanofiber-based systems involve organic solvents in the fabrication procedure^6^. Here, we used a green process, including pullulan as a food-grade biopolymer and water as the solvent. Pullulan is a linear polysaccharide produced from the fungus *Aureobasidium pullulans* and consists of maltotriose type units i.e. α-(1 → 6)-linked with (1 → 4)-α-d-triglucosides^19^. This unique linkage pattern leads to high adhesion and tensile strength of pullulan^20^. Unlike many other polysaccharides, pullulan dissolves easily in water due to the low degree of hydrogen bonding. It provides good processing potential for forming films and fibers, and due to the non-hygroscopic nature shows a considerable inter-molecular mechanical strength^8,19,21,22^. Furthermore, this biopolymer is an appropriate carrier for drug delivery applications due to its non-mutagenic, non-carcinogenic, biodegradable, mucoadhesive, and cytoadhesive properties^6,9,13^.

Since antimicrobial resistance is one of the biggest global threats to public health, and we are currently on the verge of a post-antibiotic era^23^, site-specific antimicrobial delivery is preferred. Approaches that increase the local concentration of antibiotics only in the sites where they are intended to have an effect, might be a way to reduce the development of drug resistance. Therefore, this study aimed to describe the impact of ingested streptomycin-loaded pullulan nanofibers on the intestinal bacterial community in rats compared with streptomycin delivery through drinking water. Additionally, the selective effect on an ingested resistant bacterial strain was assessed.

## Results and discussion

### Streptomycin encapsulation preserves antimicrobial properties

**Figure 1:**
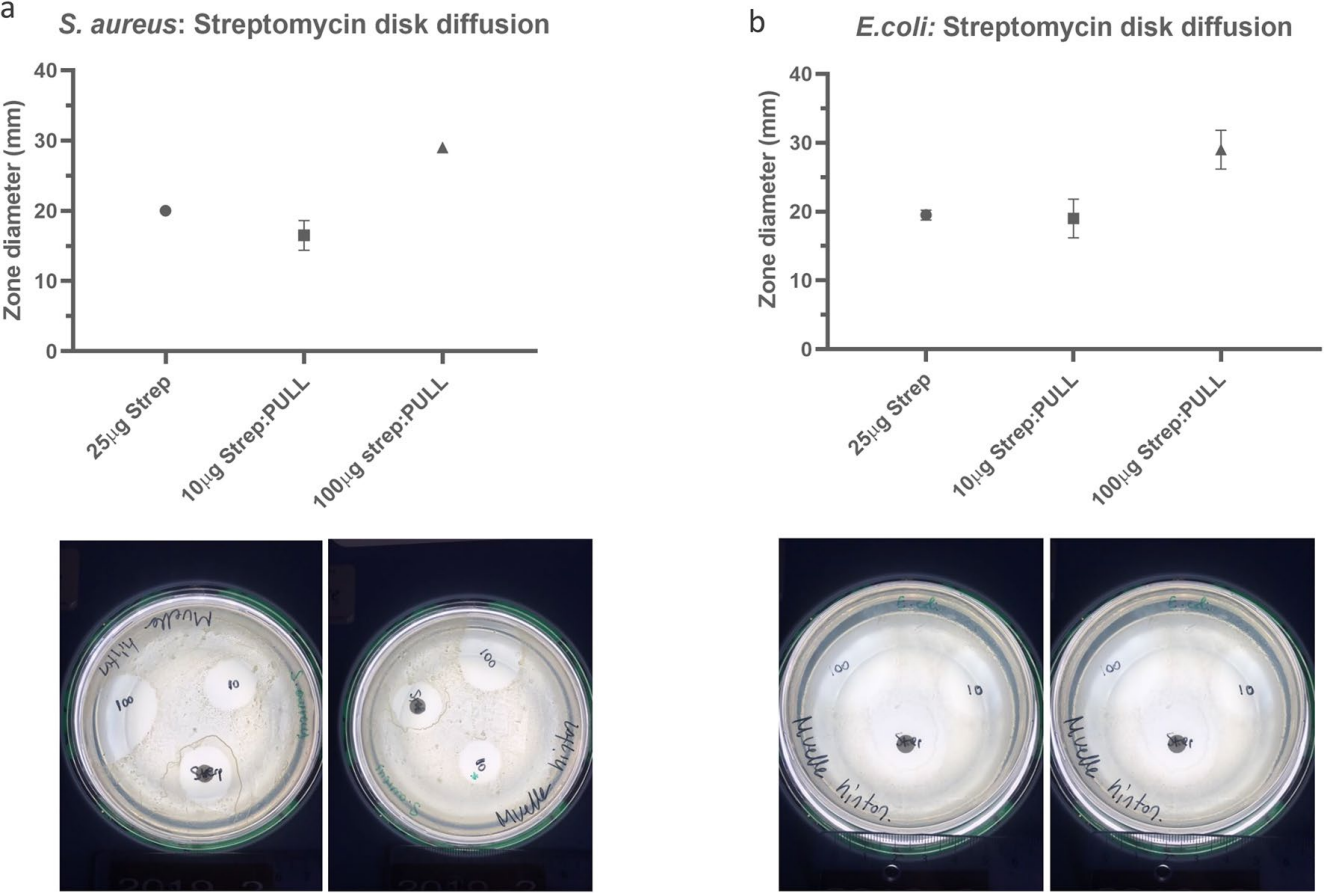
Disk diffusion assay. (**a**) *S. aureus* disk diffusion results of pullulan loaded with streptomycin and (**b**) *E. coli* disk diffusion results of pullulan loaded with streptomycin.

Disk-diffusion assays, performed by the Kirby-Bauer method, confirmed that the streptomycin antimicrobial activity remained after loading into electrospun fibers. Thus, streptomycin-loaded nanofibers inhibited the growth of sensitive *E. coli* and *S. aureus* strains (Fig. 1). Overall, we conclude that encapsulation of the drug did not affect the antimicrobial properties of the drug. Thus, electrospun nanofibers were suitable for maintaining the optimum therapeutic level of the antimicrobial drug in vitro. A similar study performed with amoxicillin has demonstrated that the nanofiber antimicrobial embedment does not compromise the release and activity of the drug^24^. Therefore, electrospun nanofibers may be relevant in various applications in the field of drug delivery.

### Streptomycin delivered in electrospun nanofibers allow proliferation of streptomycin-resistant *E. coli* in rats

**Figure 2:**
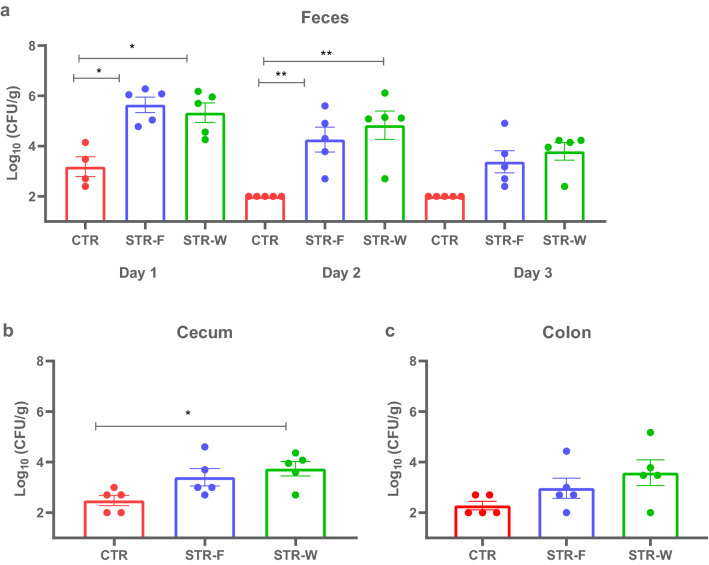
CFU counts. (**a**) *E. coli* CFU counts per gram of fecal material at Day 1, Day 2, and Day 3; (**b**) *E. coli* CFU counts per gram of cecum content at Day 1, Day 2, and Day 3; (**c**) *E. coli* CFU counts per gram of colon content at Day 1, Day 2, and Day 3. *(*P* < 0.05); **(*P* < 0.01); *** (*P* < 0.001). Only four samples were obtained from CTR on Day 1.

A rat study was performed to investigate the effects of streptomycin-carrying nanofibers on the degree of colonization of streptomycin-resistant *E. coli* MG1655-K12. Before the inoculation, no background resistance to streptomycin or gentamycin was detected in the fecal samples from these animals (data not shown). On Day 1 and Day 2 after inoculation, significantly more streptomycin-resistant *E. coli* were found in fecal samples from rats that received streptomycin either encapsulated into the nanofibers (STR-F) or in drinking water (STR-W) than in the control (CTR) group (Fig. 2a). No difference was observed between STR-F and STR-W (Fig. 2a). Higher numbers of resistant *E. coli* were found in cecal samples of the STR-W animals, while the difference between the STR-F group and the CTR was not significant (Fig. 2b,c). No difference in resistant *E. coli* numbers between any of the groups was observed in colonic and fecal samples obtained on Day 3, likely because the streptomycin treatment ceased on Day 2 in both STR-W and STR-F.

Streptomycin, given as 5 g/L in drinking water, has for decades been used in animal models to suppress facultative anaerobic bacteria, and thereby allow ingested streptomycin-resistant proteobacteria species to overcome the colonization barrier^25–27^. In the current experiment, we have applied a much lower concentration (0.25 g/L) in drinking water of the STR-W rats in an attempt to match the absolute amount of 7 mg of streptomycin given as nanofibers to the STR-F rats. Nevertheless, our results demonstrate that it is possible to obtain intestinal proliferation of resistant bacteria at this 20-fold lower concentration than the currently applied standard.

### Electrospun nanofibers delivery provides a higher concentration of streptomycin

Measurement of actual water consumption during the experiment revealed that the total streptomycin consumed by STR-W rats was around 12.5 mg, considering an estimated 50% spillage. As mentioned above, streptomycin dosed by nanofibers to STR-F rats during the experiment was approximately 7 mg.

**Figure 3:**
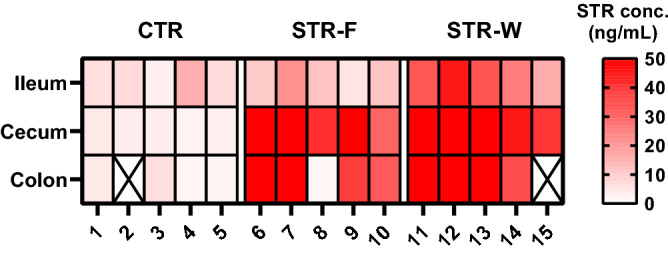
Streptomycin concentrations. Heatmap reflecting approximate levels of streptomycin present in ileum, cecum and colon STR-W and STR-F levels are different in ileal samples (P < 0.005), while levels in cecum and colon do not differ between the two groups. The assay was saturated above 50 ng/mL. Figure was made in GraphPas Prism as described in the methods section.

The concentration of streptomycin released in the gut from nanofibers was assessed by ELISA of rat intestinal content obtained on Day 3. Interestingly, we observed a significantly (P < 0.005) lower concentration of streptomycin in the ileal samples from the STR-F group, as compared to the STR-W group (Fig. 3). We speculate that the coated gelatin capsules start dissolving in the small intestine and release the electrospun pullulan fibers, which have mucoadhesive properties^9^. The pullulan nanofibers then dissolve and form a jelly-like, mucoadhesive construct, retaining the drug partially through the ileum and moving into the large intestine, where more drug get gradually released.

In line with our observations, core–shell fibers, which are produced by coaxial electrospinning, have previously shown successful sustained release of hydrophilic drugs such as streptomycin^28^. Regarding drug delivery applications using pullulan nanofibers, there are several studies focusing on fast dissolving properties of pullulan and recommending it for fast delivery approaches via the buccal route^8,19,29,30^ or for wound dressing applications^6,31^. Our findings suggest that antibiotic-loaded pullulan nanofibers are also suitable for delivery to the large intestine, due to the mucoadhesive properties of the dissolved pullulan.

### Streptomycin alters the gut microbiota composition

**Figure 4:**
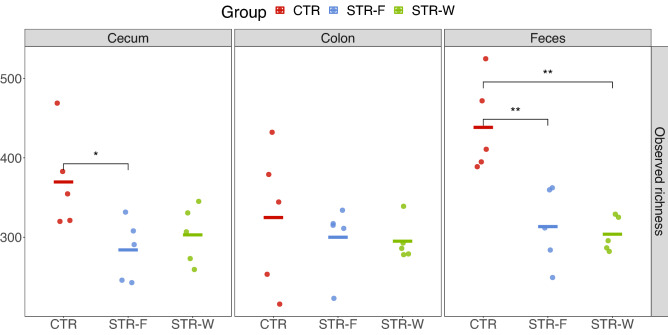
Microbial richness in cecum, colon, and feces. Samples are indicated by dots, while horisontal bars mark the averages. P-values were from the permutational test of the mean and shown as: *(*P* < 0.05); **(*P* < 0.01); ***(*P* < 0.001).

Sequencing of 16S rRNA gene amplicons was performed to assess the effect of streptomycin treatment on the intestinal microbiota. Microbial richness was significantly reduced in fecal samples from STR-F (*P* = 0.008), and STR-W (*P* = 0.007) groups, compared with the CTR group (Fig. 4). In the cecum, a slightly higher richness was observed in the CTR group than in STR-F samples.

**Figure 5:**
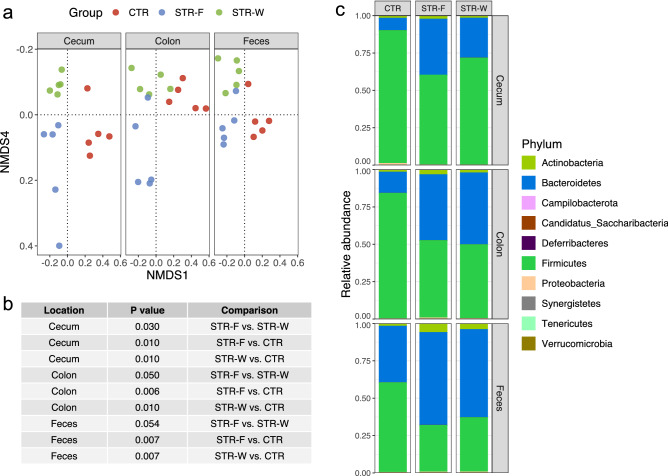
Microbial compositions in cecum, colon, and feces. (**a**) The overall microbial compositions of samples at the ASV level were assessed with and Bray–Curtis distance and visualized with non-metric multi-dimensional scaling (NMDS). (**b**) A table to show the comparison of beta diversity between groups at a location and the P-values are derived from the PERMANOVA models using Bray–Curtis distance. (**c**) Bar plots showing the taxonomic composition of top 10 most abundant Phylum.

Beta-diversity was compared between groups based on the Bray–Curtis dissimilarity and visualized with non-metric multidimensional scaling (NMDS). Three distinct clusters represented STR-W, STR-F and CTR samples, respectively (Fig. 5a). Overall, the treatment was more influential than the location in the gut, based on the PERMANOVA model (0.27 [R^2^ for Group] *vs.* 0.09 [R^2^ for location], *P* = 0, permutation test with 100 iterations). The pairwise comparison of all groups at each location was significant in all cases, except for fecal samples from the two STR-treated groups (Fig. 5b).

Together, Bacteroidetes and Firmicutes constituted more than 99% of the ASVs identified in rat samples. We found a significant reduction of the relative abundance of Firmicutes and a corresponding relative increase of Bacteroidetes in STR-W and STR-F groups in samples from cecum, colon and feces (Fig. 5c).

**Figure 6:**
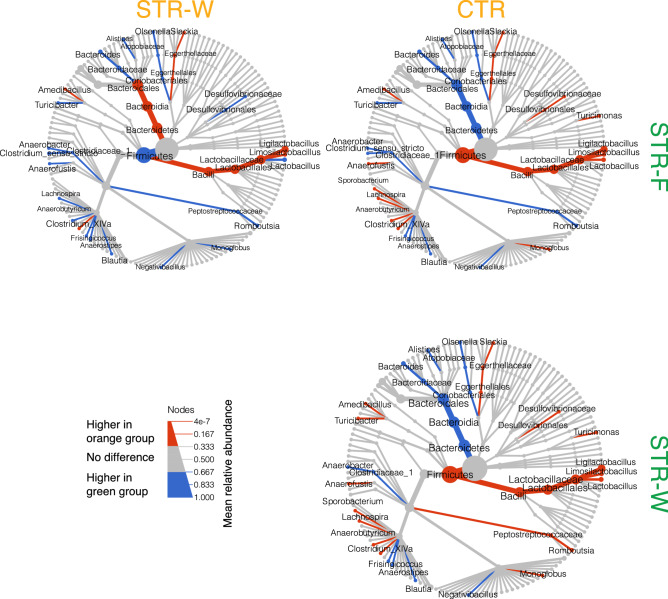
Tree plot showing the comparison of relative taxon abundances between groups. The largest node in the center is the kingdom bacteria. Along the tree branch outward, the next node is the phylum level, then followed by Class, Order, Family, and Genus. The size of the node is proportional to the mean relative abundance in the corresponding phylogenetic level. A node is labelled and colored when it has significantly different relative abundances (permutation test) between groups. Figure was made in R as described in the “Methods” section.

At family level, lower levels of *Lactobacillaceae* were found in the cecum of STR-F animals as compared to both STR-W and CTR (Fig. 6), while a higher abundance of *Peptostreptococcaceae*, specifically *Romboutsia,* was seen (Fig. 6). *Peptostreptococcaceae* are commensals in healthy rats^32^, and *Romboutsia*, which belongs to the *Peptostreptococcaceae* family, consists of anaerobe microorganisms adapted to utilize a wide range of carbohydrates, and relies on exogenous amino acids and peptides for protein synthesis^33^. We speculate that the observed higher levels of this species in the STR-F group than in the STR-W group results from the fermentation of nanofibers in the colon, which directly or indirectly promotes the growth of bacteria within this family. The indigestible portions of the nanofibers were composed of maltotriose units connected by α-1,6 bonds, which are resistant to mammalian intestinal enzymes but are available for bacterial fermentation in the large bowel^34^.

## Conclusion

Streptomycin can be incorporated into electrospun nanofibers without losing the antibacterial activity, and furthermore it can successfully be retained through the small intestine and delivered to the large intestine of rats by dosing with encapsulated nanofibers. Our results suggest that electrospun nanofibers represent an advanced alternative for the delivery of antimicrobials to the large intestine. This approach is likely to allow for reduction in the amount of antibiotics needed to be effective, and thereby to reduce the risk of development of resistance, and cause less disturbance of the indigenous microbiota. Indeed, delivery by nanofibres was seen to affect the microbiota in a different way than delivery through drinking water.

## Methods

### Fabrication of antibiotic-loaded nanofibers

Pullulan was kindly provided by HAYASHIBARA CO., LTD (Japan). Water was purified using a Milli-QPlus 185 water purification system (Millipore, Bedford, MA) with resistivity higher than 18 MΩ·cm. Streptomycin standard disks (25 µg per disk) were purchased from Oxoid, Denmark. Hexamethyldisilazane (HMDS) was obtained from Merck (Germany). Streptomycin, Gentamycin, Trehalose, and RSM (skim milk powder) were purchased from Sigma-Aldrich, Denmark.

Streptomycin was loaded into pullulan electrospun nanofibers with final concentrations of 1 and 10% respectively (w/w; weight of streptomycin to the weight of polymer). For encapsulation of streptomycin in Pullulan, streptomycin powder was first dissolved in water, and then pullulan powder was added to the solution to achieve the desired concentration. For making 1% or 10% streptomycin to Pullulan constructs (w/w), first, a streptomycin solution of 0.2% or 2% (w/v) in water was made under slight stirring at 25 ℃ for 4 h. Then pullulan powder was added to make a 20% solution (w/v) of Pullulan in water and was stirred overnight at room temperature. For instance, for fabrication of a 1% (w/w) streptomycin: pullulan nanofiber sheet, streptomycin was dissolved in water (conc. 2 mg/mL) under slight stirring at 25 ℃ for 4 h. Then pullulan powder (0.2 g) was added to the 1 mL solution to make the desired final concentration of 20% pullulan in water for optimal electrospinning. Finally, the solution was sterile-filtered through a 25 mm filter (Sigma Aldrich) and used for electrospinning.

For electrospinning, a customized conventional electrospinning system including a high voltage source (Glassman, FJ50P2.4-F22), a syringe pump (Aladdin, AL-1000) connected to a syringe with a 21 G hypodermic needle, and a static collector was used. The optimized spinning parameters were set to a feeding rate of 1 mL/h, a voltage of 14–16 kV, a needle tip-to-collector distance of 15 cm, a humidity of 35%, and a temperature of 25 °C. The electrospinning chamber including the pump and the collector was disinfected by EtOH 70% before starting each electrospinning procedure.

### In vitro antibacterial activity assay

To investigate whether the antimicrobial activity efficacy of streptomycin remains after the electrospinning, we performed a disk-diffusion assay by the Kirby–Bauer method^35^. The antibacterial activity of the streptomycin loaded into nanofiber sheets was analyzed against Gram-negative (*Escherichia coli* (*E. coli*) MG1655 subst. K12 (ATCC 47076)) and Gram-positive (*Staphylococcus aureus* (*S. aureus*) (ATCC 2928)) streptomycin sensitive bacteria. The *E. coli* and *S. aureus* strains were grown overnight in Luria Bertani plates (LB) at 37 °C. An inoculum for each sample was prepared and the turbidity of the suspension was adjusted in a densitometer (Sensititre–Nephelometer, Thermo Fisher, Denmark) in comparison with the 0.5 McFarland standard. Then, a hundred microliters of the inoculum were spread on Mueller–Hinton agar plates. The electrospun sheets containing 10 µg and 100 µg (from 1 and 10% w/w streptomycin:pullulan nanofibers) were cut with a biopsy punch of 6 mm, then placed alongside the standard Streptomycin disks of the same diameter (25 µg) (Oxoid, Denmark) as a control. Non-streptomycin-loaded electrospun sheets (pullulan nanofibers) were included as negative controls. Samples were incubated at 35 °C for 16–18 h. The diameter of the inhibition zone was measured and compared with breaking point values provided by the EUCAST guidelines (https://www.eucast.org/fileadmin/src/media/PDFs/EUCAST_files/Breakpoint_tables/v_11.0_Breakpoint_Tables.pdf).

### Antibiotic-loaded nanofibers for in vivo study

To dose rats with antibiotic-loaded nanofibers, the electrospun sheets of 10% streptomycin:pullulan were cut manually to make square particles of around 1 mm in length. Then, hard-shelled gelatine capsules (Torpac size 9) were coated in a 12% (w/v) solution of Eudragit L100 in IPA where dibutyl sebacate was added as a plasticizer in a 5% w/w ratio relative to Eudragit. Finally, capsules were filled with 12 mg of particles (equivalent to 1.2 mg streptomycin per capsule) before dosing to the rats.

### Animal experiment

**Figure 7:**
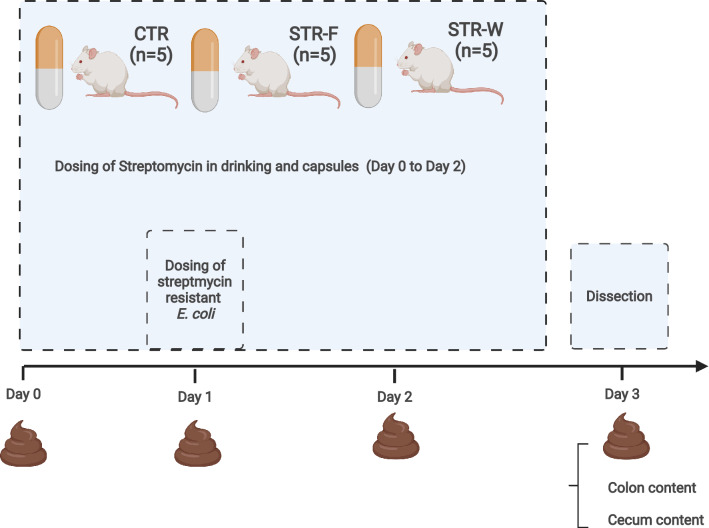
Study outline. Schematic representation of the in vivo study, representing CTR (control), which received a placebo hard-shelled gelatine capsule, STR-F (streptomycin-loaded nanofibers), which received a capsule coated with Eudragit 100S and loaded with antibiotic-loaded pullulan particles two times per day, and STR-W (streptomycin in the drinking water) received a placebo hard-shelled gelatine capsule and Streptomycin in the drinking water (0.25 g/L). All groups received a single dose of 10^8^ CFU of streptomycin-resistant *Escherichia coli*. Figure was created in BioRender by the authors.

To find out whether the nanofiber electrospun sheet loaded with streptomycin affected the establishment of streptomycin-resistant bacteria in the intestine, we performed a rat study involving oral inoculation of the animals with the streptomycin-resistant mCherry-labelled *E. coli* MG1655 subst. K12, Taxon Identifier 511145 (https://www.uniprot.org/uniprot/A0A4D6FVK6) in association with streptomycin delivered either as encapsulated nanofibers, in the drinking water or without streptomycin as control (Fig. 7). The fluorescence-labeled strain was chosen to allow easy verification by microscopy.

Fifteen male 8-weeks-old Sprague–Dawley rats (Charles River) were allocated to three groups of five and housed individually in scantainers (Scanbur). After an acclimatization period of seven days, the animals were treated with streptomycin either in drinking water (STR-W), inside of particles of electrospun nanofibers (STR-F), or did not receive any antimicrobial drug (CTR). The CTR rats received a placebo hard-shelled gelatine capsule, STR-F rats received a hard-shelled gelatine capsule filled with antibiotic-loaded pullulan particles as described above twice a day for 3 days (Day 0, 1 and 2), while the STR-W rats received an empty placebo hard-shelled gelatine capsule and Streptomycin in the drinking water (0.25 g/L) for the same three days (Fig. 7). We aimed at a concentration in drinking water, which would make the total consumption of streptomycin by the STR-W rats match the approximately 7 mg given to the STR-F rats considering that the average water consumption was between ca. 30–50 mL of water/animal/day. For this study, the streptomycin- and gentamycin-resistant *E. coli* were incubated on LB agar (SSI Diagnostica A/S) for 16 h, as described earlier. A colony was transferred to LB broth supplemented with 20 µg/mL gentamycin and 100 µg/mL streptomycin overnight/16 h at 37 °C while shaking. The culture was centrifuged at 5000×*g* for 10 min, washed and resuspended in sterile phosphate-buffered saline (PBS) to a cell density of 10^9^ CFU/mL. Twenty-four hours after the onset of streptomycin treatment, all groups received a single dose of approximately 10^8^ CFU of the strain. Animals were fed and received water ad libitum throughout the whole experiment.

### Enumeration of resistant *E. coli* in the rat gut

Fecal samples (approximately 1 g) were collected before inoculation and 24 h, 48 h, and 72 h after inoculation. After 72 h, animals were euthanized by CO_2_ asphyxiation followed by decapitation. Rats were dissected and luminal contents were aseptically collected from the distal ileum, cecum, and colon regions. The contents from these sections were weighed and homogenized. Tenfold dilutions were spread on LB agar plates supplemented with streptomycin (100 µg/mL) or gentamycin (25 µg/mL) (Sigma-Aldrich, Denmark), as appropriate. CFU counts were assessed after 24–72 h incubation at 37 °C under anaerobic conditions. Random colonies were analysed by mass spectrometry using Bruker Reflex™ III MALDI-TOF (Bruker‐Daltonik, Germany) to ensure that the isolates corresponded to *E. coli* K-12 MG1655.

### ELISA assay

To estimate the quantity of drug released in the intestinal lumen, an ELISA assay was performed to verify the concentration of streptomycin present in fecal content. Fecal pellets from the ileum, cecum, and colon were analyzed. Samples were homogenized and diluted, and the procedure was performed according to the SM Streptomycin ELISA kit (Elabscience, USA) (Catalog # E-FS-E031) manufacturer instructions. The optical density (OD) was measured using a microplate reader Biotek EL800 (Agilent, DK), set at 450 nm and 630 nm. Data were processed using GraphPad Prism Software 8.1.1.

### DNA extraction, sequencing, and bioinformatics

DNA extraction, library preparation, and subsequent sequencing on the Ion Torrent platform were performed as previously described^36^. Briefly, samples consisted of fecal samples at 72 h (n = 15), samples from ileum (n = 15) and samples from colon (n = 15). Microbial DNA was extracted using ~ 0.2 g of materials with the DNeasy® PowerLyzer® PowerSoil® isolation kit from Qiagen. The extracted DNA was stored at − 20 °C before use.

Amplification of the V3-region of the 16S rRNA gene was performed as follows: PCR mastermix consisted of 0.2 µL Phusion High-Fidelity DNA polymerase (ThermoFisher Scientific F-553L), 4 µL HF buffer, 0.4 µL dNTP (10 mM of each base), 1 µM forward primer (PBU; 5ʹA-adapter-TCAG-barcode-CCTACGGGAGGCAGCAG-3ʹ) and 1 µM reverse primer (PBR; 5ʹ-trP1-adapter-ATTACCGCGGCTGCTGG-3ʹ) and 5 ng community fecal DNA in 20 µL total reaction volume. Both primers (TAG Copenhagen A/S) were linked to sequencing adaptors and the forward primer additionally contained a unique 10 bp barcode (Ion Xpress™ Barcode Adapters) for each sample. The PCR program consisted of initial denaturation for 30 s at 98 °C, followed by 24 cycles of 98 °C for 15 s and 72 °C for 30 s, and lastly, 72 °C for 5 min to allow final extension before cooling to 4 °C. No-template controls were included for each PCR run, all resulting in less than 0.05 ng/µL. The PCR products were purified using HighPrepTM PCR Magnetic Beads (MAGBIO®, AC-60005) with a 96-well magnet stand (MAGBIO®, MyMag 96), according to the manufacturer recommendation. DNA quantity was measured using Qubit® dsDNA HS assay (InvitrogenTM, Q32851) and sequenced on chip using the Ion OneTouchTM and Ion PGM systems with a 318-Chip v2 incorporating the Hi-Q chemistry in 200 bp runs.

Raw sequences were demultiplexed with barcodes and primers removed using QIIME2^37^. The obtained sequences were further filtered to range from 125 to 180 bp. These pre-processed reads were analyzed with the DADA2 pipeline in R^38^, using the parameters recommended for Ion Torrent. The obtained amplicon sequence variants (ASVs) were thereafter taxonomically annotated using the Ribosomal Database Project (RDP) database (release 11.5).

### Statistics and reproducibility

Statistical analysis was performed with QIIME2, R, and GraphPad Prism Software 8.1.1. Samples were rarefied to an even depth of reads before calculating richness. CFU counts, streptomycin concentration, microbial richness, and taxa abundances were compared with a two-sided permutational test. ASVs (Amplicon sequencing variant) with less than 0.01% relative abundances were removed before further analysis. Beta diversity was assessed based on the Bray–Curtis distance matrix and compared with the permutational multivariate analysis of variance (PERMANOVA). The taxonomic composition shown with trees was generated with R-package “metacoder”^39^. The significance level was set at 0.5. Comparison of ELISA-assessed streptomycin levels in ceca of STR-F and STR-W animals was done by t-test assuming equal variances.

### Animal ethical statement

Danish Animal Experiments Inspectorate approved the animal trial under license number 2020-15-0201-00484. The experiment protocol was pre-registered by DTU’s BioFacility. Animals were monitored daily during the experiment, and the experiment was carried out in accordance with Danish national guidelines and overseen by the Institute’s Animal Welfare Committee for animal care and use. Methods are reported in accordance with ARRIVE guidelines.

## Data Availability

Sequence data have been deposited at NCBI Sequence Read Archive under the number PRJNA758361.

## References

[1] Nitzan O, Elias M, Peretz A, Saliba W (2016). Role of antibiotics for treatment of inflammatory bowel disease. World Journal of Gastroenterology..

[2] Francino MP (2016). Antibiotics and the human gut microbiome: Dysbioses and accumulation of resistances. Front. Microbiol..

[3] Shah T, Baloch Z, Shah Z, Cui X, Xia X (2021). The intestinal microbiota: Impacts of antibiotics therapy, colonization resistance, and diseases. Int. J. Mol. Sci..

[4] Fair RJ, Tor Y (2014). Antibiotics and bacterial resistance in the 21st century. Perspect. Med. Chem..

[5] Patra JK (2018). Nano based drug delivery systems: Recent developments and future prospects 10 Technology 1007 Nanotechnology 03 Chemical Sciences 0306 Physical Chemistry (incl. Structural) 03 Chemical Sciences 0303 Macromolecular and Materials Chemistry 11 Medical and He. J. Nanobiotechnol..

[6] Ajalloueian F (2022). Amoxicillin-loaded multilayer pullulan-based nanofibers maintain long-term antibacterial properties with tunable release profile for topical skin delivery applications. Int. J. Biol. Macromol..

[7] Mamun MM, Sorinolu AJ, Munir M, Vejerano EP (2021). Nanoantibiotics: Functions and properties at the nanoscale to combat antibiotic resistance. Front. Chem..

[8] Hsiung E (2022). Antibacterial nanofibers of pullulan/tetracycline-cyclodextrin inclusion complexes for fast-disintegrating oral drug delivery. J. Colloid Interface Sci..

[9] Bulman SE (2015). Pullulan: A new cytoadhesive for cell-mediated cartilage repair. Stem Cell Res. Ther..

[10] Hong L (2019). Pullulan nanoparticles as prebiotics enhance the antibacterial properties of *Lactobacillus plantarum* through the induction of mild stress in probiotics. Front. Microbiol..

[11] Mir M, Ahmed N, UrRehman A (2017). Recent applications of PLGA based nanostructures in drug delivery. Colloids Surf. B Biointerfaces.

[12] Jeckson TA, Neo YP, Sisinthy SP, Gorain B (2021). Delivery of therapeutics from layer-by-layer electrospun nanofiber matrix for wound healing: An update. J. Pharm. Sci..

[13] Ajalloueian F (2022). Multi-layer PLGA-pullulan-PLGA electrospun nanofibers for probiotic delivery. Food Hydrocoll..

[14] Liu X (2022). Electrospun core (HPMC–acetaminophen)–Shell (PVP–sucralose) nanohybrids for rapid drug delivery. Gels.

[15] Liu H (2022). Hybrid films prepared from a combination of electrospinning and casting for offering a dual-phase drug release. Polymer.

[16] Zhao K (2021). Modified tri-axial electrospun functional core–shell nanofibrous membranes for natural photodegradation of antibiotics. Chem. Eng. J..

[17] Ji Y, Song W, Xu L, Yu DG, Bligh SWA (2022). A review on electrospun poly(amino acid) nanofibers and their applications of hemostasis and wound healing. Biomolecules.

[18] Fahimirad S, Ajalloueian F (2019). Naturally-derived electrospun wound dressings for target delivery of bio-active agents. Int. J. Pharm..

[19] Ponrasu T, Chen B-H, Chou T-H, Wu J-J, Cheng Y-S (2021). Fast dissolving electrospun nanofibers fabricated from jelly fig polysaccharide/pullulan for drug delivery applications. Polymers (Basel).

[20] Bailore NN, Balladka SK, Doddapaneni SJDS, Mudiyaru MS (2021). Fabrication of environmentally compatible biopolymer films of pullulan/piscean collagen/ZnO nanocomposite and their antifungal activity. J. Polym. Environ..

[21] Singh RS, Saini GK, Kennedy JF (2008). Pullulan: Microbial sources, production and applications. Carbohydr. Polym..

[22] Celebioglu HU (2017). Mucin- and carbohydrate-stimulated adhesion and subproteome changes of the probiotic bacterium *Lactobacillus acidophilus* NCFM. J. Proteom..

[23] Irwin R (2020). Imagining the postantibiotic future: The visual culture of a global health threat. Med. Hum..

[24] Wang S (2012). Encapsulation of amoxicillin within laponite-doped poly(lactic-co-glycolic acid) nanofibers: Preparation, characterization, and antibacterial activity. ACS Appl. Mater. Interfaces.

[25] Lasaro M (2014). *Escherichia coli* isolate for studying colonization of the mouse intestine and its application to two-component signaling knockouts. J. Bacteriol..

[26] Poulsen LK, Licht TR, Rang C, Krogfelt KA, Molin S (1995). Physiological state of *Escherichia col*i BJ4 growing in the large intestines of streptomycin-treated mice. J. Bacteriol..

[27] Nevola JJ, Stocker BAD, Laux DC, Cohen PS (1985). Colonization of the mouse intestine by an avirulent *Salmonella typhimurium* strain and its lipopolysaccharide defective mutants. Infect. Immun..

[28] Sohrabi A, Shaibani PM, Etayash H, Kaur K, Thundat T (2013). Sustained drug release and antibacterial activity of ampicillin incorporated poly (methyl methacrylate) e nylon6 core/shell nano fibers. Polymer (Guildf.).

[29] Vila MMDC, Tardelli ER, Chaud MV, Tubino M, Balcão VM (2014). Development of a buccal mucoadhesive film for fast dissolution: Mathematical rationale, production and physicochemical characterization. Drug Deliv..

[30] Qin ZY, Jia XW, Liu Q, Kong BH, Wang H (2019). Fast dissolving oral films for drug delivery prepared from chitosan/pullulan electrospinning nanofibers. Int. J. Biol. Macromol..

[31] Román JT (2019). Pullulan nanofibers containing the antimicrobial palindromic peptide LfcinB (21–25)Pal obtained: Via electrospinning. RSC Adv..

[32] Leng Y (2016). Effects of acute intra-abdominal hypertension on multiple intestinal barrier functions in rats. Nat. Publ. Gr..

[33] Gerritsen J (2017). Genomic and functional analysis of *Romboutsia ilealis* CRIB T reveals adaptation to the small intestine. PeerJ..

[34] Spears JK, Karr-Lilienthal LK, Fahey GC (2005). Influence of supplemental high molecular weight pullulan or γ-cyclodextrin on ileal and total tract nutrient digestibility, fecal characteristics, and microbial populations in the dog. Arch. Anim. Nutr..

[35] Hudzicki J (2016). Kirby-Bauer Disk Diffusion Susceptibility Test Protocol.

[36] Laursen MF (2016). Infant gut microbiota development is driven by transition to family foods independent of maternal obesity. mSphere.

[37] Bokulich NA (2018). Optimizing taxonomic classification of marker-gene amplicon sequences with QIIME 2’s q2-feature-classifier plugin. Microbiome.

[38] Callahan BJ (2016). DADA2: High-resolution sample inference from Illumina amplicon data. Nat. Methods.

[39] Foster ZSL, Sharpton TJ, Grünwald NJ (2017). Metacoder: An R package for visualization and manipulation of community taxonomic diversity data. PLoS Comput. Biol..

